# Plastid Located WHIRLY1 Enhances the Responsiveness of *Arabidopsis* Seedlings Toward Abscisic Acid

**DOI:** 10.3389/fpls.2012.00283

**Published:** 2012-12-24

**Authors:** Rena Isemer, Kirsten Krause, Nils Grabe, Nobutaka Kitahata, Tadao Asami, Karin Krupinska

**Affiliations:** ^1^Institute of Botany, Christian-Albrechts-University of KielKiel, Germany; ^2^Department of Arctic and Marine Biology, University of TromsøTromsø, Norway; ^3^Department of Applied Biological Chemistry, The University of TokyoTokyo, Japan

**Keywords:** abscisic acid, abamine, *Arabidopsis*, dual localization, *gun*, plastid signaling, salicylic acid, WHIRLY1

## Abstract

WHIRLY1 is a protein that can be translocated from the plastids to the nucleus, making it an ideal candidate for communicating information between these two compartments. Mutants of *Arabidopsis thaliana* lacking WHIRLY1 (*why1*) were shown to have a reduced sensitivity toward salicylic acid (SA) and abscisic acid (ABA) during germination. Germination assays in the presence of abamine, an inhibitor of ABA biosynthesis, revealed that the effect of SA on germination was in fact caused by a concomitant stimulation of ABA biosynthesis. In order to distinguish whether the plastid or the nuclear isoform of WHIRLY1 is adjusting the responsiveness toward ABA, sequences encoding either the complete WHIRLY1 protein or a truncated form lacking the plastid transit peptide were overexpressed in the *why1* mutant background. In plants overexpressing the full-length sequence, WHIRLY1 accumulated in both plastids and the nucleus, whereas in plants overexpressing the truncated sequence, WHIRLY1 accumulated exclusively in the nucleus. Seedlings containing recombinant WHIRLY1 in both compartments were hypersensitive toward ABA. In contrast, seedlings possessing only the nuclear form of WHIRLY1 were as insensitive toward ABA as the *why1* mutants. ABA was furthermore shown to lower the rate of germination of wildtype seeds even in the presence of abamine which is known to inhibit the formation of xanthoxin, the plastid located precursor of ABA. From this we conclude that plastid located WHIRLY1 enhances the responsiveness of seeds toward ABA even when ABA is supplied exogenously.

## Introduction

WHIRLY1 is a DNA binding protein shown to be located both in chloroplasts and the nucleus and to have the same molecular weight in both compartments (Grabowski et al., 2008). After its synthesis on 80S ribosomes, WHIRLY1 is targeted to chloroplasts where it is processed by cleavage of an N-terminal plastid transit peptide (PTP). The functionality of the PTP has been shown before by subcellular detection of a WHIRLY1:GFP fusion protein and by import assays (Krause et al., 2005). Recently, it has been shown that recombinant hemagglutinin (HA)-tagged WHIRLY1 synthesized in plastids of transplastomic tobacco plants is translocated to the nucleus where it activates transcription of pathogen response (*PR*) genes (Isemer et al., 2012). This suggests that WHIRLY1 is sequestered in plastids before it gets translocated to the nucleus to adjust gene expression (Krause and Krupinska, 2009). Although the stimuli that lead to its translocation are still unknown, this feature makes WHIRLY1 an ideal candidate for transducing information from plastids to the nucleus.

The plastid and the nuclear isoforms of WHIRLY1 were shown to be responsible for diverse unique functions in the two compartments. Plastid located WHIRLY1 has been found in stroma fractions and additionally was shown to associate with plastid DNA (Pfalz et al., 2006; Majeran et al., 2012) and with plastid RNA (Prikryl et al., 2008; Melonek et al., 2010). WHIRLY1 from *Arabidopsis thaliana* has been implicated in plastid genome repair, assigning the protein an important role in maintaining plastid genome stability (Maréchal et al., 2009; Cappadocia et al., 2010). This plastid specific function of WHIRLY1 is also accomplished by WHIRLY3, the second member of the WHIRLY protein family that can be found in plastids (Maréchal et al., 2009; Cappadocia et al., 2010). Furthermore, WHIRLY1 was shown to promote the splicing of intron containing plastid encoded RNAs in maize and barley (Prikryl et al., 2008; Melonek et al., 2010). The nuclear form of WHIRLY1 has been implicated in PR reactions in potato as well as in *A. thaliana* (Desveaux et al., 2000, 2004, 2005). Transcriptional activation of *PR* genes by WHIRLY1 was reported to depend on salicylic acid (SA; Desveaux et al., 2000, 2004). SA is the only plant hormone which is completely and exclusively synthesized inside plastids (Wildermuth et al., 2001). Besides its well-known roles in pathogen defense signaling, it has many other functions in addition. For example, SA has been implicated in acclimation to high light and in regulation of the redox homeostasis (Mateo et al., 2006; Mühlenbock et al., 2008).

In recent years, plastids as sites of SA biosynthesis have also been implicated in perception of pathogens and in innate immunity (Padmanabhan and Dinesh-Kumar, 2010). For example, elicitors of the bacterial plant pathogen *Pseudomonas syringae* were predicted to localize to chloroplasts (Guttman et al., 2002). In tobacco plants infected by tobacco mosaic virus (TMV) the chloroplast located protein N-receptor interacting protein 1 (NRIP1) was shown to mediate innate immunity. Even more interestingly, the chloroplast protein is recruited by a viral effector to the cytoplasm and nucleus (Caplan et al., 2008) indicating that redistribution of regulatory proteins from chloroplasts to the nucleus is involved in plant responses toward pathogens. Very recently, changes in the level of calcium within plastids and formation of singlet oxygen were shown to precede the stimulation of SA biosynthesis in response to pathogens (Nomura et al., 2012).

With regard to its formation inside plastids, SA could be the trigger inducing the translocation of WHIRLY1 from plastids to the nucleus. Such a mechanism would be in accordance with the model on WHIRLY1 activation during pathogen defense proposed by Brisson and co-workers (Desveaux et al., 2000, 2005). This model suggested that an inactive pool of WHIRLY1 is activated upon an increase in the level of SA and thus is able to bind to promoters of *PR* genes. It is feasible that the WHIRLY1 pool, which is inactive with regard to activation of the target genes, is identical with the subset of plastid located WHIRLY1.

Salicylic acid has also been found to be involved in regulation of plant development. In *Arabidopsis*, the level of SA is highly elevated during early seedling development (Preston et al., 2009) which might be related to the proposed role of SA in establishing defense mechanisms during germination (Rajjou et al., 2006). Application of SA was shown to inhibit germination and it has been proposed that this inhibitory effect is due to a concomitant increase in ABA (Rajjou et al., 2006) as measurements in wheat seedlings suggested (Shakirova et al., 2003). The stimulation of ABA synthesis might occur inside plastids, which harbor the key enzyme of ABA biosynthesis, 9-cis-epoxycarotenoid dioxygenase (NCED) that converts carotenoids into xanthoxin. Xanthoxin leaves the plastid and is converted to ABA in the cytosol (Cutler et al., 2010; Seo and Koshiba, 2011). Indeed, plants deficient in accumulation of SA were also shown to have lower levels of ABA making it difficult to analyze the effects of one or the other hormone originating from plastids separately (Abreu and Munné-Bosch, 2009). Accumulating evidence suggests that ABA is associated with plastid signaling. Indeed, ABA responsive *cis*-elements (ABREs) have been found in the promoters of many target genes of plastid signals (Rook et al., 2006; Koussevitzky et al., 2007). Moreover, the transcription factor ABI4, which was first described in connection with ABA insensitivity (Finkelstein, 1994), was shown to be common to different pathways of plastid signaling. Accordingly, the *abi4* mutant was found to be insensitive toward plastid signals produced during conditions of impaired chloroplast development (Koussevitzky et al., 2007).

In this study we analyzed the impact of WHIRLY1 on the responsiveness of seeds toward the plastid originating hormones SA and ABA during germination. *WHIRLY1* T-DNA insertion mutants (*why1*) of *Arabidopsis* were shown to be insensitive toward SA as well as ABA. When the biosynthesis of ABA was inhibited by abamine known to prevent formation of the ABA precursor xanthoxin, SA had no effect on germination. This showed that SA only indirectly affects seedling development by stimulation of ABA biosynthesis. Opposite to the mutants and to transgenic plants accumulating WHIRLY1 exclusively in the nucleus, transgenic plants accumulating WHIRLY1 in both plastids and the nucleus showed hypersensitivity toward ABA during germination. To conclude, our data indicate that WHIRLY1 enhances the responsiveness of seeds toward ABA only when it is located in the plastids. The plastid located precursor of ABA, xanthoxin, is not involved in this response, because germination was inhibited by external ABA also in the presence of the biosynthesis inhibitor abamine.

## Materials and Methods

### Plant material and growth conditions

Plants of *A. thaliana*, ecotype Columbia, were grown under controlled conditions at 21°C with 13 h of illumination at a light intensity of 100 μmol photons s^−1^ m^−2^. Under these conditions plants developed flowers within 8–9 weeks and mature seeds could be harvested after 12–14 weeks. T-DNA insertion lines Salk_023713 (*why1-1*) and Salk_147680 (*why1-2*) for *WHIRLY1* (At1g14410) were purchased from NASC and the positions of the T-DNA insertions were confirmed by PCR with the primers suggested by the T-DNA express tool of the SALK institute (http://signal.salk.edu/tdnaprimers.2.html). The absence of the initial ATG codon in the *WHIRLY1* mRNA of *why1-1* and *why1-2*, respectively, was shown by RT-PCR using the WHY1_fwd and WHY1_rvs primers. The *ACTIN2* gene was amplified to show equal amounts of templates. All primer sequences are listed in Table A1 of the Appendix. The seeds of the *gun1-1* and *gun1-102* lines (Voigt et al., 2010) were a gift of Dario Leister. The seeds of the *gun5* line (Susek et al., 1993) were a gift of Bernhard Grimm.

### Germination assays

*Arabidopsis* seeds were surface sterilized as described before (Aronsson and Jarvis, 2011) and put on full-strength Murashige and Skoog (MS) medium (Duchefa, Haarlem, The Netherlands) containing 1.5% (w/v) sucrose and 1% (w/v) phyto agar (Duchefa, Haarlem, The Netherlands). After imbibition at 4°C in the dark over night, plates were exposed to controlled growth conditions at 21°C with 16 h of illumination at a light intensity of 60 μmol photons s^−1^ m^−2^ for germination assays in the presence of ABA. For germination assays in the presence of SA a neutral filter was used to eliminate UV radiation from the light spectra which reduced the light intensity to 30 μmol photons s^−1^ m^−2^. Seeds of comparable age and storage conditions were used and the same amounts of medium were filled into the plates. To determine phenotypical differences during germination, sterile seeds were put on rectangular plates standing upright and photographed every day. The impact of phytohormones on germination was assayed as described (Choy et al., 2008). For preparation of stock solutions the sodium salt of SA (Sigma-Aldrich, Steinheim, Germany) was dissolved in sterile water to a final concentration of 1 M. ABA (6 cis-trans; Sigma A1049, Sigma-Aldrich, Steinheim, Germany) was first dissolved in 1 ml of 1 M NaOH and then diluted with sterile deionized water to a final stock concentration of 25 mM. Abamine (Han et al., 2004) was dissolved in dimethylsulfoxide to a final stock concentration of 130 mM. Twenty to 50 seeds each were plated on media containing the different compounds. Seven days after imbibition the number of seeds, where emerging radicles could be observed with the naked eye, was determined.

### Construction of transgenic lines overexpressing *WHIRLY1*

The full-length coding sequence of *AtWHIRLY1* and the truncated *AtWHIRLY1* sequence lacking the first 141 bp encoding the plastid transit peptide (PTP) were amplified by PCR using the cDNA *U10139*, cloned into pENTR/TOPO gateway vector and sequenced to verify PCR product sequences. The HA tag was included in frame in the sequence of the reverse primer. After transfer of the two different *AtWHIRLY1* constructs into the binary destination vector pB2GW7.0 (Karimi et al., 2002), *why1-1* plants were transformed using *Agrobacterium tumefaciens* mediated flower transformation employing vacuum infiltration (Bechtold and Pelletier, 1998). Successfully transformed plants were selected by their resistance toward 0.1% (w/v) glufosinate ammonium (Basta, Bayer Crop Science, Germany) which was applied by spraying.

### Preparation of subcellular fractions and immunological gel blot analyses

Chloroplasts were prepared from leaves of 7-week-old *Arabidopsis* plants and purified on percoll step gradients as described (Gruissem et al., 1986). Nuclear proteins were isolated from frozen leaves of 7-week-old *Arabidopsis* plants as described by Busk and Pagès (1997). Proteins were separated under denaturing conditions on 14% (w/v) acrylamide gels, transferred to nitrocellulose membranes and immunodecorated using standard protocols as described (Isemer et al., 2012). A monoclonal antibody directed toward the HA tag was purchased from Roche Diagnostics GmbH (Mannheim, Germany). Antibodies against the cytochrome *b*_559_ apoprotein A (Vallon et al., 1987) and RNA polymerase I (RNA POL-I; Agrisera, Vännäs, Sweden) respectively, were used to monitor the purity of the nuclear and chloroplast fractions.

## Results

### Characterization of two *WHIRLY1* mutants of *Arabidopsis thaliana*

**Figure 1:**
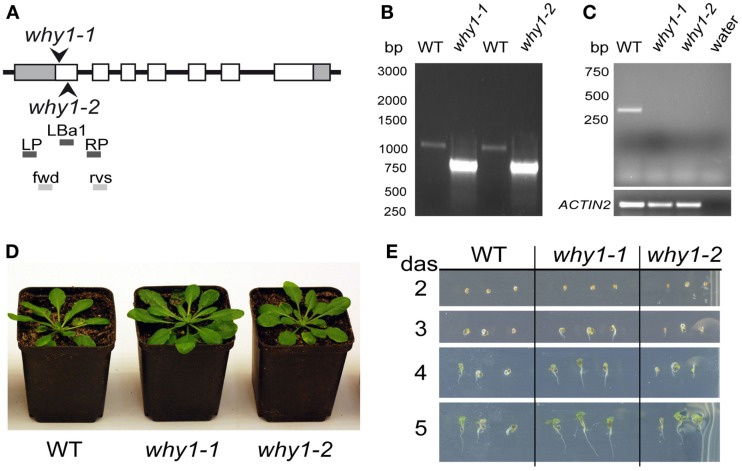
**Characterization of *why1* mutants**. **(A)** Scheme showing the positions of T-DNA insertions in two independent mutant lines (*why1-1* and *why1-2*) for the *WHIRLY1* gene. The sites of the T-DNA insertions are indicated by arrowheads. Additionally, the positions of primers used for homozygous screening (LP, LBa1, RP) and verification of the presence of the initial start codon ATG in the *WHIRLY1* mRNA (fwd, rvs) are shown. **(B)** Genomic DNA derived from wildtype, *why1-1* and *why1-2* lines, respectively, was used to test for homozygosity of the mutants by use of the primers shown in **(A)**. **(C)** In order to detect the presence of *WHIRLY1* mRNA in the *why1-1* and *why1-2* lines, respectively, cDNA derived of the two mutant lines and wildtype (WT) and the primers shown in **(A)** were used in a RT-PCR. Primers specific for *ACTIN2* were used for loading controls. **(D)** Phenotype of the *why1-1* and *why1-2* mutants was compared to wildtype (WT) when grown under standard growth conditions. Six-week-old plants are shown. **(E)** Germination of *why1-1* and *why1-2* was compared to the wildtype (WT) under standard growth conditions. The phenotype of the seedlings was monitored 2–5 days after sowing (das).

Previously, functions of WHIRLY1 have been connected with the phytohormone SA (Desveaux et al., 2000, 2004, 2005; Xiong et al., 2009). Therefore, the initial aim of this study was to investigate whether WHIRLY1 (At1g14410) plays a role in SA induced inhibition of germination in *Arabidopsis*. For this purpose the two independent T-DNA insertion lines *why1-1* (Salk_023713) and *why1-2* (Salk_147680) were used. In the genomic DNA of mutant *why1-1* the T-DNA insertion was found to be located directly after the starting codon ATG, whereas in the *why1-2* line it was found to be located 25 base pairs further downstream (Figure 1A). Homozygosity of *why1-1* and *why1-2*, respectively, was shown by PCR (Figure 1B) with primers LP, RP, and LBa1 as suggested by the T-DNA express tool of the SALK institute (http://signal.salk.edu/tdnaprimers.2.html). In the mutants the LP-RP amplification product of the wildtype allele (approximately 1050 bp, see Figure 1B) was not visible. Only the smaller amplificate corresponding to the T-DNA insertion was visible, indicating that both mutant lines are indeed homozygous. The primers “fwd” and “rvs” spanning the AUG start codon, exon 1 and part of exon 2 (see Figure 1A) were used to probe for the presence of *WHIRLY1* mRNA. A 300-bp band corresponding to that region was detected only in wildtype plants whereas the control mRNA for ACTIN2 was detectable in wildtype as well as in mutants (Figure 1C). When cultivated under standard growth conditions the phenotypes of *why1-1* and *why1-2* mutants were indistinguishable from the wildtype (Figures 1D,E). This observation was true for mature plants (Figure 1D) as well as for young seedlings inspected at different times after germination on agar plates containing MS medium (Figure 1E). These findings are in accordance with previous results on the *why1-1* and *why1-2* mutant lines (formerly named KO-1 and KO-2; Yoo et al., 2007).

### *WHIRLY1* mutant seeds show reduced responsiveness to salicylic acid as well as to abscisic acid

**Figure 2:**
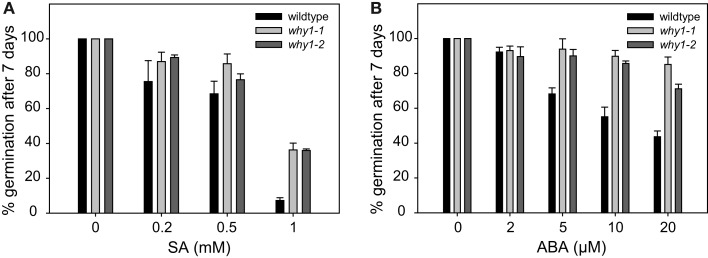
**Inhibition of germination by the phytohormones salicylic acid and abscisic acid**. **(A)** In order to investigate the sensitivity of the two *why1* mutant lines (*why1-1* and *why1-2*) to the inhibitory effect of salicylic acid (SA) on germination, mutant, and wildtype seeds were grown on agar medium containing 0.2–1 mM SA. **(B)** The effect of abscisic acid (ABA) on germination of *why1* mutant seeds and wildtype seeds was scored by growth on agar medium containing 2–20 μM ABA. For germination assays in the presence of either SA or ABA, respectively, the percentage of seeds showing radicle emergence was scored after 7 days of growth at 21°C in a 16-h light/dark regime. Values are the means ± SEM obtained from at least three independent experiments with 20–50 seeds each. The averaged percentage of germinated seeds in the control experiments without SA and ABA, respectively, was set to 100% and used as a reference to calculate the percentage of germination in the assays performed in the presence of SA or ABA.

Germination of *Arabidopsis* seeds is known to be inhibited by SA (Rajjou et al., 2006). In order to investigate whether this biological activity of SA is dependent on WHIRLY1, the two independent *WHIRLY1* T-DNA insertion mutants *why1-1* and *why1-2* were germinated on agar medium in the presence of different concentrations of SA ranging from 0.2 to 1 mM. For comparison, the same experiment was performed with wildtype seeds. The germination rate was scored 7 days after imbibition of seeds by assessing with the naked eye the number of seeds with emerged radicles. At concentrations ranging from 0.2 to 0.5 mM SA, germination of the *why1* mutant seeds was 10–15% more efficient than of wildtype seeds (Figure 2A). The most prominent alteration of germination was visible at a concentration of 1 mM, when the germination rate in the mutants was almost 40%, compared to an almost complete failure of the wildtype to germinate (Figure 2A).

External addition of SA to barley seeds was found to induce a transient increase in the level of abscisic acid (ABA; Shakirova et al., 2003), and the inhibitory effect of SA on germination of *Arabidopsis* seeds was assigned to the activity of ABA (Rajjou et al., 2006). Hence, it is possible that the tendentiously reduced sensitivity of the *why1* mutants toward SA might in fact be due to a reduced responsiveness toward ABA. Germination assays were therefore also performed in the presence of different concentrations of ABA ranging from 2 to 20 μM. Ten micromolars of ABA were sufficient to inhibit germination of almost 50% of the wildtype seeds. Twenty micromolars of ABA lowered the germination rate of the wildtype seeds to 40% whereas the germination rate in the mutant was on average twice as high (Figure 2B), illustrating that *why1* seeds are less sensitive to the inhibitory effect of ABA on germination.

### The inhibitory effect of SA on germination is abolished by abamine, an inhibitor of abscisic acid biosynthesis

**Figure 3:**
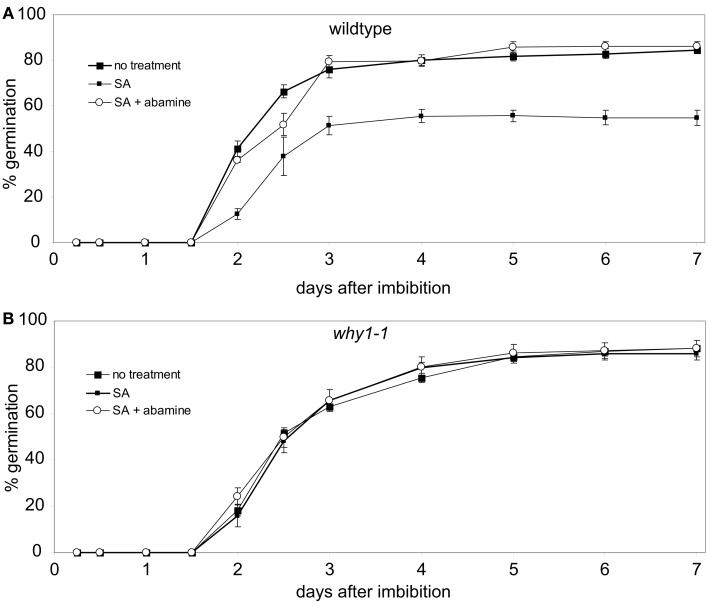
**Effects of abamine on salicylic acid mediated repression of germination**. Wildtype seeds **(A)** and seeds of the *why1* mutant **(B)** were grown on agar medium containing 500 μM salicylic acid (SA) or 500 μM SA + 50 μM abamine, respectively. Germination rates of the two treatment conditions were compared to untreated seeds during the first 7 days after imbibition. Values are the means ± SEM obtained from five independent experiments with 20–50 seeds each.

To investigate whether the inhibitory effect of SA on germination under the conditions employed here is indeed caused by a concomitant increase in the level of ABA, germination of wildtype seeds on SA containing agar plates during the first 7 days of development was assayed in the presence of abamine. Abamine is a specific inhibitor of the enzyme NCED preventing the formation of the ABA precursor xanthoxin (Han et al., 2004) which is the last intermediate of the ABA biosynthetic pathway being produced in the plastid (Seo and Koshiba, 2011). When wildtype seeds were treated with 0.5 mM SA, less than 20% of seeds were able to germinate in the first 2 days after imbibition (Figure 3A). The germination rate did not increase further after 4 days at which time it had reached 80% (Figure 3A). When wildtype seeds were exposed to SA in the presence of abamine, germination was observed to be as efficient as under control conditions without addition of SA (Figure 3A). When the same assays were done with seeds of the *why1-1* mutant line, no differences in germination efficiency were detectable between seeds treated either with only SA or with SA and abamine together, respectively, and the control setup (Figure 3B).

### *why1-*, *gun*1-, and *gun*5 mutants share the same insensitivity of germination toward salicylic acid and abscisic acid

**Figure 4:**
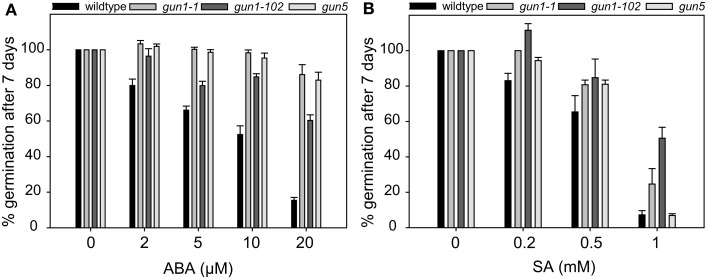
**Inhibition of germination by abscisic acid and salicylic acid in *gun* mutants**. In order to investigate the germination of *gun* mutants in the presence of abscisic acid (ABA) **(A)** and salicylic acid (SA) **(B)**, respectively, seeds of the well described *gun* mutants *gun1-1*, *gun1-102*, and *gun5* were grown on agar medium containing 2–20 μM ABA or 0.2–1 mM SA. The percentage of seeds showing radicle emergence was scored after 7 days of growth at 21°C in a 16-h light/dark regime. Values are the means ± SEM obtained from at least three independent experiments with 20–50 seeds each. The average percentage of germination of seeds in the control experiments without ABA or SA, respectively, was set to 100% and used as a reference to calculate the percentage of germination in the germination assays done in the presence of ABA or SA.

Germination assays revealed that *why1* mutants are insensitive toward SA and also to ABA. The responsiveness of *why1* observed in this study is in the same range as the responsiveness of the *abi1* mutant (Finkelstein and Somerville, 1990) indicating that WHIRLY1 might play a role in the ABA signaling pathway. Due to its dual localization in plastids and the nucleus, WHIRLY1 is an excellent candidate protein for transducing plastid signals to the nucleus. Interestingly, seeds of some *genomes uncoupled* (*gun*) mutants, that are characterized by impaired plastid-to-nucleus signaling, were likewise shown to be insensitive toward ABA (Choy et al., 2008; Cottage et al., 2010; Voigt et al., 2010; Kerchev et al., 2011). To investigate whether *why1* mutants are comparable to *gun* mutants with regard to ABA sensitivity of seeds, seeds of two *gun1* mutants (*gun1-1* and *gun1-102*), and one *gun5* mutant were subjected to the germination assay in the presence of different concentrations of ABA. At a concentration of 20 μM of ABA at which less than 20% of wildtype seeds were observed to germinate (Figure 4A), 60% of *gun1-102* seeds, and about 80% of *gun1-1* as well as *gun5* seeds showed germination (Figure 4A). This shows that the insensitivity of *gun1* and *gun5* mutants toward ABA is comparable to that of the *why1* mutants.

By application of the ABA inhibitor abamine it became obvious that the inhibitory effect of SA on germination is actually due to a concomitant increase in ABA content (Figure 3). Hence, mutants that are insensitive toward ABA during germination should also be insensitive toward SA. In order to investigate whether this is also true for the *gun* mutants, seeds of *gun1-1*, *gun1-102*, and *gun5* as well as wildtype seeds were placed on agar medium in the presence of different concentrations of SA ranging from 0.2 to 1 mM. While less than 70% of the wildtype seeds did germinate at a concentration of 0.5 mM SA, germination of the *gun* mutant seeds was only reduced by 20% by these concentrations (Figure 4B). The insensitivity of the *gun* mutants toward SA is in the same range as the insensitivity of the *why1* mutants (Figure 2A).

### The plastid isoform of WHIRLY1 enhances the responsiveness of seedlings toward abscisic acid

**Figure 5:**
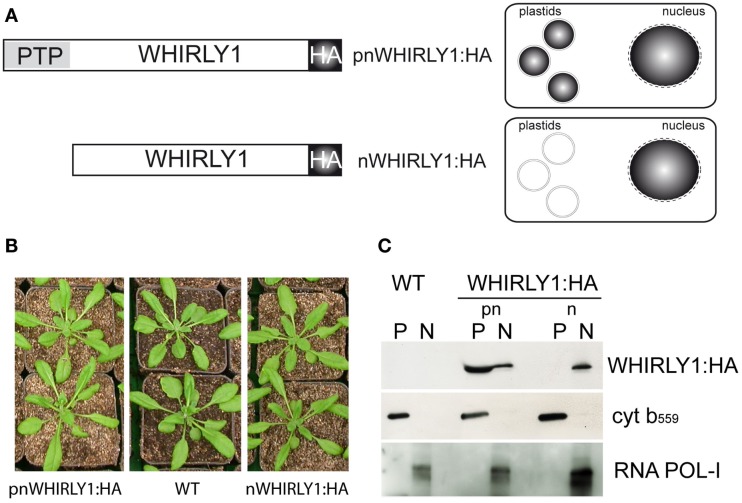
**Characterization of transgenic lines possessing HA-tagged versions of either the complete *WHIRLY1* gene (pnWHIRLY1:HA) or a truncated form lacking the plastid transit peptide (PTP) sequence (nWHIRLY1:HA)**. **(A)** Schematic drawings of constructs used for transformation and the expected subcellular distribution of the HA-tagged WHIRLY1. HA, hemagglutinin tag. **(B)** Phenotypes of the pnWHIRLY1:HA and nWHIRLY1:HA overexpression lines was compared to wildtype (WT) at 5 weeks after sowing. **(C)** Immunoblot analysis of protein extracts derived from chloroplasts (P) and nuclei (N) of the wildtype (WT) and transgenic lines overexpressing constructs for two HA-tagged versions of WHIRLY1 (pnWHIRLY1:HA and nWHIRLY1:HA). An antibody specific for the HA-tag was used to detect recombinant WHIRLY1 (WHIRLY1:HA). No HA-tag signal could be detected in wildtype (WT). Purity of the fractions was shown by immunodetection of cytochrome *b*_559_ (cyt *b*_559_) as marker for chloroplasts and RNA polymerase I (RNA POL-I) as marker for the nucleus.

**Figure 6:**
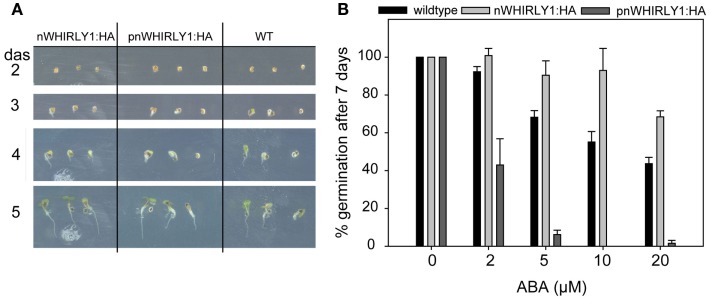
**Inhibition of germination by abscisic acid in transgenic lines overexpressing WHIRLY1**. **(A)** Germination of pnWHIRLY1:HA and nWHIRLY1:HA overexpression lines was compared to the wildtype (WT) under standard growth conditions. The phenotype of the seedlings was monitored 2–5 days after sowing (das). **(B)** Transgenic lines overexpressing the sequences encoding the HA-tagged versions of WHIRLY1 were included in the abscisic acid (ABA) germination assay in order to show dose response of ABA inhibition of germination. The percentage of seeds showing radicle emergence was scored after 7 days of growth at 21°C in a 16-h light/dark regime. Values are the means ± SEM obtained from at least three independent experiments with 20–50 seeds each. The averaged percentage of germinated seeds in the control experiments without ABA was set to 100% and used as a reference to calculate the percentage of germination in the assays performed in the presence of ABA.

Germination assays indicated that WHIRLY1 is enhancing the responsiveness of seeds toward ABA. To investigate whether either WHIRLY1 in plastids or WHIRLY1 in the nucleus or both isoforms are involved in the ABA response, the *WHIRLY1* gene was overexpressed under control of the *35S* promoter in the *why1-1* mutant background. Plants of a line accumulating an HA-tagged full-length protein (pnWHIRLY1:HA) were compared with a line accumulating a truncated protein lacking the PTP (nWHIRLY1:HA; Figure 5A). Both of the overexpression lines did not show any phenotypical difference when compared to wildtype (Figures 5B and 6A).

The subcellular localization of the two recombinant proteins that is shown schematically in Figure 5A was confirmed by immunoblot analyses with protein extracts prepared from isolated chloroplasts and nuclei, respectively. In plants overexpressing the complete *WHIRLY1*-HA construct, the HA-tag was detectable in both compartments. In comparison, the protein arising from a construct devoid of the PTP accumulated exclusively in the nucleus (Figure 5C). Antibodies directed against cytochrome *b*_559_ as a marker for chloroplasts and against RNA POL-I as a marker for nuclear proteins, respectively, were used to monitor the purity of both fractions.

To investigate the responsiveness of the transgenic lines possessing WHIRLY1:HA toward ABA, germination assays were performed under the same conditions as used above for *why1* mutants. In the absence of ABA no differences in germination were observed between wildtpye seeds and transgenic seeds (Figure 6A). When ABA was applied, seeds of plants accumulating WHIRLY1 in the nucleus only (nWHIRLY1:HA) were less sensitive to ABA than wildtype seeds (Figure 6B). They are hence comparable to the seeds of *why1* mutants (Figure 2B). Seeds of plants accumulating WHIRLY1, however, in both chloroplasts and nuclei (pnWHIRLY1:HA) displayed a much higher responsiveness to ABA than wildtype seeds (Figure 6B). At a concentration of 5 μM ABA less than 10% of the seeds were observed to germinate whereas more than 60% of wildtype seeds germinated (Figure 6B). With a second independent pnWHIRLY1:HA line similar results were obtained.

### ABA inhibits germination in the presence of abamine

**Figure 7:**
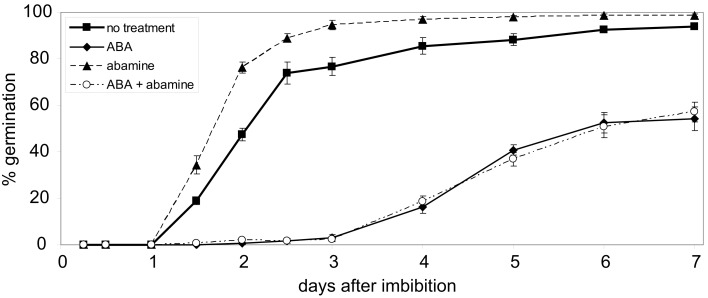
**Effect of abamine on abscisic acid mediated repression of germination**. Wildtype seeds were grown on agar medium containing either no additive or 10 μM abscisic acid (ABA), 50 μM abamine or 10 μM ABA + 50 μM abamine, respectively. Germination rates were determined in the period from 1 to 7 days after imbibition. Values are the means ± SEM obtained from five independent experiments (20–50 seeds each).

The results of the germination assays performed with seeds of transgenic lines overexpressing the full-length WHIRLY1:HA (pnWHIRLY1:HA) or a truncated version lacking the plastid transit peptide (nWHIRLY1:HA) indicated that the plastid located isoform of WHIRLY1 is enhancing the responsiveness of seeds toward ABA. This is surprising considering that ABA has been exogenously applied and even endogenous ABA is formed from xanthoxin outside of plastids (Cutler et al., 2010; Seo and Koshiba, 2011). In order to investigate whether the effect of ABA is mediated by changes in the level of xanthoxin inside plastids, the effect of ABA on germination of wildtype seeds was assayed in the presence of abamine which is known to inhibit the enzyme NCED catalyzing the formation of xanthoxin (Han et al., 2004). Addition of abamine enhanced the rate of germination indicating that the inhibitor indeed prevented the formation of ABA (Figure 7). When ABA was added alone the number of germinating seeds was reduced by 50% (Figure 7). When the same concentration of ABA was applied in the presence of abamine, the germination rate was the same as that determined in the presence of ABA only (Figure 7). This result shows that the exogenously applied ABA inhibits germination independent of the intrinsic production of the ABA precursor xanthoxin in plastids.

## Discussion

The WHIRLY1 protein was first discovered as a nuclear transcription factor implicated in *PR* and its function as a nuclear activator of *PR* genes was shown to depend on SA (Desveaux et al., 2000). Later, WHIRLY1 was found to be imported into plastids (Krause et al., 2005) whereas its potential to be translocated from the plastid to the nucleus was not known until recently (Isemer et al., 2012). SA is the only plant hormone completely synthesized in plastids (Wildermuth et al., 2001). It is hence possible that activation of WHIRLY1 in response to pathogens in fact occurs inside the organelle and not in the nucleus. This idea would be in accordance with the model presented by Brisson and co-workers on activation of an inactive pool of WHIRLY1 by SA (Desveaux et al., 2004, 2005). This would mean that plant responses toward pathogens mediated by SA would be under control of plastids as also recently suggested (Nomura et al., 2012).

The results obtained in this study suggested that WHIRLY1 is also involved in SA induced inhibition of germination. By simultaneous addition of SA and abamine, an inhibitor of the biosynthesis of ABA, it was, furthermore, demonstrated that the effect of SA on germination can be explained by its stimulatory effect on the biosynthesis of ABA. Although ABA is formed from xanthoxin in the cytoplasm, the enzymes limiting the level of its precursor are located inside chloroplasts (Seo and Koshiba, 2011). The crosstalk between ABA and SA was observed to be important in determining the outcome of plant pathogen interactions (Cao et al., 2011). The levels of both hormones and also that of jasmonic acid, another stress related hormone originating from plastids, were shown to depend on each other (Abreu and Munné-Bosch, 2009). It is likely that there is a crosstalk of the three stress related hormones completely or partially synthesized inside plastids which are the sites where stress is primarily perceived in the plant cell (Bouvier et al., 2009).

The germination assays clearly demonstrated that WHIRLY1 is enhancing the responsiveness of germinating seeds toward ABA. Hence, the *why1* mutants also belong to the group of *aba insensitive* (*abi*) mutants. The previously identified *abi* mutants *1-5* were selected for their ability to germinate in the presence of 10 μmol ABA (Finkelstein, 1994), a concentration inhibiting germination of wildtype seeds. ABA responsiveness of the *why1* mutants is comparable to the *abi1* mutant (Finkelstein and Somerville, 1990) and even lower than the ABA sensitivity of the *abi4* and *abi5* mutants (Finkelstein, 1994). WHIRLY1 is found both in plastids and in the nucleus of the same cell (Grabowski et al., 2008). Germination assays with transgenic lines accumulating WHIRLY1 either in both plastids and the nucleus (pnWHIRLY) or only in the nucleus (nWHIRLY1) showed that it is the plastid localized WHIRLY1 that enhances the responsiveness of seedlings toward ABA. The inhibitory effect of ABA on germination was observed after external addition of ABA to the medium as well as after induction of its biosynthesis by addition of SA. It was shown before that WHIRLY1 as well as the other members of the WHIRLY protein family can form 24-mers in plastids, thereby building up a hollow oligomer which might store metabolites in order to protect the cell from possibly toxic compounds (Cappadocia et al., 2012). Such structures could also act as scaffolds binding specific proteins involved in signaling and thereby controlling the flow of signaling information (Zeke et al., 2009). Furthermore, the results obtained by use of the overexpression lines demonstrate an important function for the plastid in adjusting the activity of ABA.

Abscisic acid was recently reported to bind to plastid located magnesium-protoporphyrin IX chelatase which was proposed to function as a receptor of ABA (Shang et al., 2010). So far, it is not known whether ABA binds to the enzyme inside plastids or to a domain of the H subunit protruding into the cytoplasm (Shang et al., 2010). Both scenarios are possible because ABA can penetrate membranes in its protonated form and is known to diffuse inside the cell where it accumulates at sites of low pH (Seo and Koshiba, 2011).

ABA responsive *cis*-elements have been found in the promoters of many genes responding also to plastid signals. Multiple copies of ABRE (ACGT, CGTGTC) known to confer ABA responsiveness are in close proximity to G-boxes which are responsible for light-induced induction of the target genes. The transcription factor ABI4, which was first described in connection with ABA insensitivity (Finkelstein, 1994), was shown to be common to different pathways of plastid signaling (Koussevitzky et al., 2007). The *ABI4* gene encodes the apetala 2 transcription factor involved in transcriptional repression of photosynthesis associated nuclear genes when chloroplast development is blocked by addition of norflurazon (NF; Koussevitzky et al., 2007). NF blocks the production of chlorophyll due to its inhibitory effect on carotenoid biosynthesis and is commonly used as a herbicide (Sandmann and Böger, 1989). Alternatively, chloroplast development has been frequently blocked by treatment of seedlings with lincomycin, an inhibitor of plastid translation (Koussevitzky et al., 2007).

Mutants impaired in plastid signaling were named *gun*. Originally, five *gun* mutants (*gun1-5*) were identified in a screen for mutants that expressed photosynthesis associated nuclear genes when seedlings were grown in the presence of NF (Susek et al., 1993; Mochizuki et al., 2001). The *abi4* mutant shows derepression of *LHCB* gene expression levels after treatment of seedlings with lincomycin and is therefore also a *gun* mutant. In contrast, in the *abi1-3* and *abi5* mutants the expression of this gene was repressed to a similar extent as in the wildtype (Koussevitzky et al., 2007). This indicates that ABI4 is involved in plastid signaling and suggests that ABA itself is a plastid signal. Germination assays with *gun* mutants *gun1-1*, *gun1-102*, and *gun5* performed under the same conditions as with the *why1* mutant showed that these mutants have reduced sensitivity toward ABA during germination, furthermore indicating an important role for ABA during plastid retrograde signaling.

Most plastid signals so far described accumulate inside the plastid, e.g., reactive oxygen species, intermediates of tetrapyrrole biosynthesis or proteins such as GUN1 (Beck, 2005; Woodson and Chory, 2008; Leister, 2012). The ABA precursor xanthoxin is formed from carotenoids inside plastids but the last steps of ABA biosynthesis take place in the cytoplasm (Seo and Koshiba, 2011). Germination assays in the presence of both ABA and abamine demonstrated that ABA itself and not the plastid located precursor xanthoxin inhibits germination. Earlier on it was deduced that ABA is unlikely to be a plastid signal because the ABA deficient mutant *aba1* failed to accumulate *LHCB* mRNA when grown in the presence of lincomycin (Koussevitzky et al., 2007). However, this conclusion is only relevant for plastid signaling induced by lincomycin and for only one of the many photosynthesis associated nuclear genes as target.

In fact, many more factors have been shown to affect plastid functionality and to change the expression of nuclear genes encoding chloroplast proteins (Leister, 2005). Plastid signals do not only control chloroplast development, but are also required for optimization of photosynthesis under changing environmental conditions to which mature plants are exposed (Pogson et al., 2008). Moreover, plastid signals were shown to affect processes not directly involving plastids such as the accumulation of anthocyanins (Ruckle and Larkin, 2009), the circadian clock (Hassidim et al., 2007), and pathogen response (Nomura et al., 2012). In fact, the entire development of plants seems to be controlled by plastids at least to some extent (Inaba and Ito-Inaba, 2010).

Taken together, the results of the studies on *why1* mutants and transgenic lines accumulating WHIRLY1 either in plastids and the nucleus or in the nucleus only suggest that the plastid located WHIRLY1 enhances the responsiveness of seeds toward ABA. Whether WHIRLY1 directly interacts with ABA and whether it is translocated from plastids to the nucleus in response to ABA remains to be investigated. In order to place WHIRLY1 within the network of plastid-to-nucleus signaling, nuclear gene expression in the *why1* mutants and in double mutants affected in addition in one of the well-known *gun* genes needs to be systematically analyzed in response to various treatments known to impair chloroplast development.

## Conflict of Interest Statement

The authors declare that the research was conducted in the absence of any commercial or financial relationships that could be construed as a potential conflict of interest.

## Appendix

**Table A1:** **Gene specific primers that were used in this study**.

Gene name	Accession no.	Primer name	Sequence
*WHIRLY1*	At1g14410	LP	5′-TGACCAACAAACTGTTGATGG-3′
		RP	5′-TCGAATGACCCACGTAAAATC-3′
		WHY1_fwd	5′-AGGGAACAAACACAAAGCGA-3′
		WHY1_rvs	5′-CAGACTGATGTTTTGAGATACAACC-3′
Left border primer for T-DNA		LBa1	5′-TGGTTCACGTAGTGGGCCATCG-3′
*ACTIN2*	At3g18780	ACTIN2_for	5′-CTAAGCTCTCAAGATCAAAGGC-3′
		ACTIN2_rev	5′-AACATTGCAAAGAGTTTCAAGG-3′
